# Hand Injury among Patients Visiting Emergency Department in a Tertiary Care Centre: A Descriptive Cross-sectional Study

**DOI:** 10.31729/jnma.7969

**Published:** 2023-01-31

**Authors:** Anurag Singh Thapa, Shankar Man Rai, Kiran Kishor Nakarmi, Bishal Karki, Mangal Gharti Magar, Krishna Kumar Nagarkoti, Peeyush Dahal, Niran Maharjan, Pashupati Babu Pokharel, Apar Lamichhane

**Affiliations:** 1 Department of Burns, Plastic and Reconstructive Surgery, National Academy of Medical Sciences, Kathmandu, Nepal; 2 Department of Burns, Plastic and Reconstructive Surgery, Kirtipur Hospital, Kathmandu, Nepal

**Keywords:** *finger injuries*, *hand injuries*, *occupational injuries*

## Abstract

**Introduction:**

The hand is a complex organ responsible for activities of daily living, making it susceptible to injuries and accidents. Hand injuries can result in significant functional impairment and it occurs in a younger productive age group. Therefore, it is important to understand the prevalence and patterns of hand injuries. The aim of the study was to find out the prevalence of hand injuries among patients visiting the emergency department of a tertiary care centre.

**Methods:**

A descriptive cross-sectional study was in the Emergency Department of a dedicated trauma center from 1 June 2022 to 31 August 2022. Ethical approval was obtained from the Institutional Review Board (Reference number: 148412078179). Demographic profile, pattern, and mechanism of hand Injuries of all 96 consecutive patients were assessed after taking informed consent. Convenience sampling method was used. Point estimate and 95% Confidence Interval were calculated.

**Results:**

Among 4679 patients visiting the emergency department of the trauma center, hand injuries were seen in 96 (2.05%) (1.64-2.46, 95% Confidence Interval).

**Conclusions:**

The prevalence of hand injuries was found to be lower than other similar studies done in similar settings.

## INTRODUCTION

The hand is a complex organ with intricate anatomy, essential for employment, communication, expression, and activities of daily living making it susceptible to injuries and accidents. Hand Injuries account for 6.6 to 28.6% of injuries.^1^ In Nepal, hand injuries accounted for 4.19% of emergency visits in the 20 to 29 year age group, representing injuries to the most productive population.^2^ It is reported that 58.5% of hand injuries had residual functional impairment.^1^ It can lead to prolonged time off work.^3^

With increasing investment in manufacturing and the mechanization of agriculture, it is essential to understand the prevalence and patterns of hand injuries in Nepal, particularly in the context of a dedicated public trauma hospital in Nepal. The study will form a baseline for further studies.

The aim of the study was to find out the prevalence of hand injuries among patients visiting the emergency department of a tertiary care centre.

## METHODS

The descriptive cross-sectional study was carried ampong patients presenting to the Emergency Department of the National Trauma Center between 1 June 2022 to 31 August 2022. Ethical approval for the study was taken from the Institutional Review Board of the National Academy of Medical Sciences, Bir Hospital (Reference number: 148412078179). Informed consent was taken from all patients and their guardians in cases of minors. The study included all patients presenting with hand injuries to the emergency of the national trauma center and excluded the patients who refused to be part of the study. Convenience sampling method was used. The sample size was calculated using the following formula:


$$\begin{array}{ll} \text{n} & ={\text{Z}}^{2}\times \frac{\text{p}\times \text{q}}{{\text{e}}^{\text{2}}}\\ & =1.96^{2}\times \frac{0.50\times 0.50}{0.02^{2}}\\ & =2401 \end{array}$$

Where,

n = minimum required sample size
Z = 1.96 at 95% of Confidence Interval (CI)
p = prevalence taken as 50% for maximum sample size calculation
q = 1-p
e = margin of error, 2%

The minimum required sample size was However, the final sample size taken was 4679.

The demographics of the patient like name, age, gender, weight, occupation, hospital number, address, time, and mode of injury along with the place of injury, hand dominance, and time duration of injury at presentation were recorded by filling the Pro-forma. The hand injury was evaluated. The description of the wound and whether the dominant hand was involved were noted. The hand was assessed for injuries to the skin and soft tissue, muscles and tendons, nerves and vessels, and bones and joints and entered into the proforma. Operative and radiographic findings were noted and the hand injury severity was classified using the Hand severity score.^4^ Hand injuries were classified based on different occupation of the patients as per the International Standard Classification of Occupations.^5^

Data collected were entered and analyzed using IBM SPSS Statistics version 25. Point estimate and 95% Confidence Interval were calculated.

## RESULTS

Among 4679 patients visiting the emergency department of the trauma center, hand injuries were seen in 96 (2.05%) (1.64-2.46, 95% CI). The mean age was 29.77±13.83 years with a range from 1 to 72 years. Most of the patients were male, i.e., 76 (79.17%) compared to females who were 20 (20.83%) in number. Right-hand dominance was seen in 87 (90.62%) of the patients while the left was seen in 6 (6.25%). Three infants and children (ages 12, 24, and 24 months) did not show hand dominance. The dominant hand was involved in 48 (50%) of the cases.

Majority of injuries occurred among patients with craft and related trade occupation 25 (26.04%) (Table 1).

**Table 1:** Occupational distribution of hand injuries (n= 96).

Occupation	n (%)
Managers	7 (7.29)
Professionals	6 (6.25)
Technicians and associate professionals	2 (2.08)
Services and sales	4 (4.17)
Skilled in agriculture, forestry, and fishery	5 (5.20)
Craft and related trade	25 (26.04)
Plant and Machine operators	1 (1.04)
Elementary occupations	20 (20.83)
Housewife	6 (6.25)
Students	17 (17.71)
Others (children and infants)	3 (3.12)

It was seen that 48 (50%) of the injuries occurred in theworkplace (Table 2). A total of 31 (32.29%) at home,and 17 (17.71%) at the roadside. Themajority of theinjuries were self-funded, i.e., 71 (73.96%) while theremaining 26 (26.04%) were funded by the employeror a second party.

**Table 2:** Activity during hand injury (n = 96).

Injury type	n (%)
Work-related	48 (50)
Leisure and sports related	3 (3.12)
Household activity related	18 (18.75)
RTA	10 (10.41)
Physical assault	4 (4.17)
Other (self-inflicted injuries like punching a mirror, windowpane, and wardrobe glass or slashing of the wrist, etc.)	13 (13.54)

Machinery was responsible for the majority of hand injuries. Road traffic accidents on a motorbike were another important cause of hand injury. Interestingly, a large number of people injured their hands while cleaning the motorbike chain while leaving the engine running (Table 3).

**Table 3:** Characteristic features observed (n= 96).

Parameters	n (%)
**Machinery**	**33 (34.37)**
Circular electric saw	18 (18.75)
Pipe cutting saw	1 (1.04)
Cement mixer	4 (4.17)
Textile machine	1 (1.04)
Sewing machine	1 (1.04)
Bag cutting machine	1 (1.04)
Jewelry making machine	1 (1.04)
Printing machine	2 (2.08)
Food processor	1 (1.04)
Meat processor	1 (1.04)
Juice pressing machine	1 (1.04)
Beetle nut (supari) cutting machine	1 (1.04)
Leisure Activity	5 (5.21)
Crushed by a bamboo pole during a festival (Jatra)	1 (1.04)
Crushed by a metal rod while playing	1 (1.04)
Crushed by a fan blade at a party	1 (1.04)
Crushed by a school bench	1 (1.04)
Sports	1 (1.04)
**Agriculture, Gardening and other Household Injury**	**11 (11.46)**
Avulsion and amputation by a rope used to tie a buffalo during feeding	1 (1.04)
Crushed by thresher for Grass cutting	1 (1.04)
Cut by a sickle while cutting grass,vegetation, gardening	5 (5.21)
Cut by knife while cutting meat, vegetables, coconut	4 (4.17)
**Construction Site and Loading-Related Injury**	**12 (12.50)**
Crushed by Metal drum	1 (1.04)
Crushed by cement block, stone block, stone slab	7 (7.29)
Crushed by stone roller	1 (1.04)
Crushed by the tractor door rod	2 (2.08)
Tin Roof Edge laceration	1 (1.04)
**Road Traffic Accident and Vehicle Related**	**18 (18.75)**
RTA Motorbike accident	9 (9.38)
Crushed by the Bus door	1 (1.04)
Crushed by motorbike chain	7 (7.29)
Crushed by a vehicle tyre	1 (1.04)
**Physical Assault and Self Harm**	**13 (13.54)**
Knife (Khukuri) Assault	4 (4.17)
Cut by a shard of glass, glass slab, striking a mirror	9 (9.38)
**Crushed by Door Hinge**	**3 (3.12)**
**Dog Bite**	**1 (1.04)**

Hand injuries can have a wide variety of presentations and patterns, with lacerations covering the majority that is 61 (63.54% ). However, most of the injuries were noted to be minor in severity.

**Table 4:** Pattern of hand Injury (n = 96).

Pattern of Hand Injury	n (%)
Left Hand	43 (44.79)
Right Hand	53 (55.21)
Dominant Hand	48 (50)
Index Finger	28 (29.17)
Long Finger	23 (23.96)
Ring Finger	16 (16.67)
Small Finger	9 (9.38)
Thumb	16 (16.67)
Dorsum of the hand/digit	53 (55.21)
Volar aspect of the hand/digit	54 (56.25)
Skin loss	21 (21.88)
Laceration	61 (63.54)
Flexor Tendon Injury	32 (33.33)
Extensor Tendon Injury	40 (41.67)
Nerve Injury	30 (31.25)
Vascular Injury	29 (30.21)
Phalangeal Fracture	40 (41.67)
Metacarpal Fracture	3 (3.12)
Carpal Fracture	1 (1.04)
Intrinsic Muscle injury	3 (3.12)
Amputations	17 (17.71)
Level of Amputation	
Distal Interphalangeal Joint	1 (1.04)
Thumb Interphalangeal Joint	1 (1.04)
Proximal Phalanges	2 (2.08)
Middle Phalanges	3 (3.12)
Distal Phalanges	9 (9.38)
Wrist	1 (1.04)
Nail Bed Injury	22 (22.92)

The mean and standard deviationof the hand injurityseverity score was 39.1 ±80.4. Majority of the injuriesthat is 40 (41.67%) were minor inseverity followed bymoderate type 19 (19.79%) (Table 5).

**Table 5:** Hand Injury Severity Score (n= 96).

Grading of severity	n (%)
Minor	51 (53.12)
Moderate	25 (26.04)
Major	15 (15.62)
Severe	5 (5.22)

## DISCUSSION

The prevalence of hand injury in this study was 2.05% which is less to the other retrospective study done in Nepal where the rate of hand injury was 4.19% of the emergency registrations.^2^ The study from Poland estimated hand injuries to occur 5.74 per 10,000 population. A systematic review of global trends in hand and wrist trauma estimates an increasing trend in hand injury by 25% particularly in low-middle and middle sociodemographic countries.^6^

It is particularly relevant as the mean age group in this study population was 29.8, which is an active working age group. The mean age group in our study is similar to the findings from the previous study from Nepal where the mean age was 28.79 for males and 30.43 for females.^7^ The ages of 20 to 30 had the highest rate of hand injuries.^2^ This is in contrast to the findings from the study done in Poland where the mean age was 37 years. The study population is again older in comparison to the study conducted in Nepal, where the mean age was 23.5. The findings of hand injury in this productive population have important repercussions. Without access to proper surgical management and long-term rehabilitation, the potential for long-term pain and loss of productivity is great.

The most common occupation to suffer hand injuries in this study were craft and trade-related workers which included mechanics, carpenters, aluminum frame workers, grill makers, and textile and garment workers. Elementary occupations like manual laborers, factory workers, and stone masons were the next most commonly injured population. Most injuries occurred in the workplace during a work-related activity. However, in the previous study from Nepal road traffic accidents were the most common cause of hand injuries.^2^ Machinery injury which includes an electrical circular saw for metal and wood cutting was the most common cause of injury which is similar to the previous studies in Nepal.^7^ Road traffic accidents were also an important cause of hand injuries similar to previous studies but it is interesting to note that a considerable number of people injured their hands while cleaning the motorbike chain with the engine running.^8^ While burn injuries to the hand and their sequelae played a major part in the surgical workload in a previous study from Nepal, burn injuries to the hand were not seen in this series.^9^

Hand injuries can have a varied presentation ranging from lacerations in the skin to amputations, causing significant functional impairment. Most of the cases in this series presented tendon injuries and phalangeal fractures. Worryingly, digital amputations were also very common in this series. While most of the patients had minor injuries as per the Hand Injury Severity score, about 14% had major to severe hand injuries.^4^ This is concerning because there was a significant correlation between the severity of hand injury and persistent disability from the polish study, where an average of 58.5% of the patients had functional hand impairment. They also demonstrated a correlation between loss of hand function and return to the preinjury profession.^1^ In a Dutch study, it was seen that the median time to return to work was 10.5 weeks with 9% taking longer than a year to return to work.^10^ Several patients had crush injuries and amputations in this series, it is worrying because it was seen in a previous study by Wong that severe crush had the highest time off work.^3^ As most of our cases belonged to crafts, trade-related and elementary professions, this can place a significant financial burden and may result in a loss of livelihood.

While most of the cases involved manual labor, only 25% of the hand injuries were funded by the employer. Most studies find that indirect costs contribute to the majority of healthcare costs. Most of the economic impact of hand injury stems from the loss of productivity.^1^

Although this is a single-center hospital-based study in a dedicated trauma center in Nepal, it is of short duration and it may not reflect the actual burden of the disease. Long-term follow-up looking into the outcome of hand injuries would be necessary to assess the impact of hand injuries.

## CONCLUSIONS

The prevalence of hand injuries was found to be lower than other similar studies done in similar settings. Workplace injury was the most common cause of hand injury. Hand Injuries occurred in a young and productive population with machinery being an important causative factor. It is essential to explore safety measures at the workplace as most of the hand injuries occurred during work-related activity in a productive age group.
